# Assessment of Physical Properties and Potential Elemental Leaching from Bauxite Tailing-Based Alkali-Activated Materials

**DOI:** 10.1021/acsomega.5c08633

**Published:** 2026-01-06

**Authors:** Igor Alexandre Rocha Barreto, Marcondes Lima da Costa

**Affiliations:** Program for Post-Graduation in Geology and Geochemistry, Institute of Geosciences, Federal University of Pará, Belém, Pará 66075-110, Brazil

## Abstract

Alkali-activated materials (AAMs) are emerging materials with enhanced properties, including high compressive strength, low shrinkage, adjustable curing rates, resistance to acids and fire, and low thermal conductivity. These materials are synthesized by reacting aluminosilicate-rich precursors with highly alkaline solutions. Given these advantageous characteristics, this study investigates the synthesis of AAMs using bauxite washing clay from the Amazon region. Specimens were prepared with bauxite washing clay and a 1.3 M NaOH solution. The raw material was characterized by using X-ray diffraction (XRD), thermal analysis (TGA-DSC), Fourier transform infrared spectroscopy, and X-ray fluorescence. The specimens were further analyzed by XRD and tested for compressive strength, water absorption, apparent porosity, and acid leaching using HNO_3_. The bauxite washing clay sample primarily comprises aluminum- and silica-rich minerals such as kaolinite and gibbsite, making it a suitable precursor for alkali activation. The synthesized specimens demonstrated low water absorption (18.61% ± 0.25) and significant mechanical strength (25.83 MPa ± 1.33). Regarding the potential release of metals and metalloids, the highest concentrations were observed for Al, Si, and Na - key elements in geopolymer formation.

## Graphical abstract

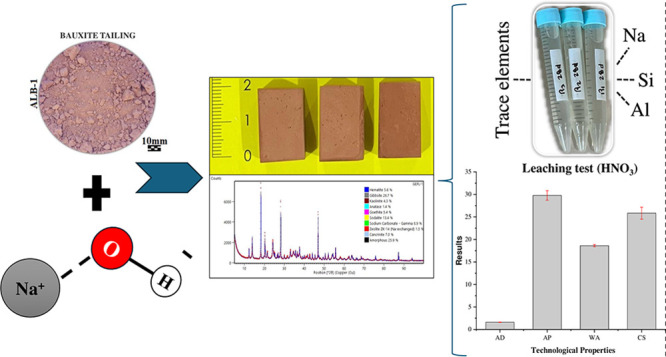

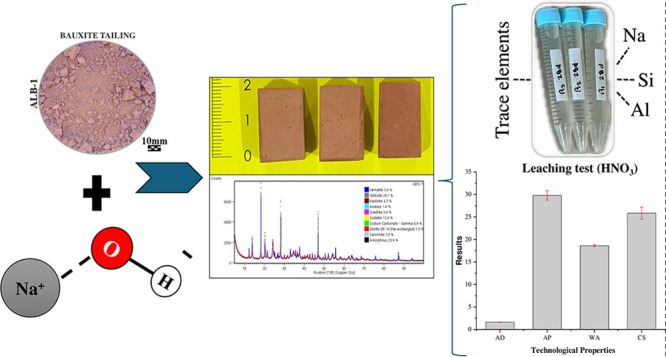

## 1 Introduction

Portland cement remains the most widely used material in the construction industry. However, its production is a major contributor to CO_2_ emissions, raising serious environmental concerns. The cement sector alone accounts for approximately 7–8% of global carbon dioxide emissions. Despite these issues, Portland cement possesses unique properties that make it difficult to be replaced.^1,2^ In recent years, alkali-activated materials (AAMs) have emerged as promising alternatives. These materials are synthesized by reaction with aluminosilicate precursors and highly alkaline solutions. AAMs exhibit several advantageous properties, including thermal and mechanical resistance, chemical resistance (e.g., to acidic environments), high compressive and flexural strength, low shrinkage, and the ability to cure at ambient temperature.^3^

The properties of AAMs are highly dependent on the type of raw materials used in their synthesis.^3^ The first material to be evaluated is the aluminosilicate source, which is mainly responsible for achieving an adequate SiO_2_/Al_2_O_3_ ratio. This ratio directly influences the final strength of the alkali-activated product. Many materials are currently being used as aluminosilicate sources, such as fly ash, glass waste, mining residues, clays, soils, synthetic reagents, and other types of waste.^4–13^ Combining different aluminosilicate sources is a common strategy to attain a more appropriate SiO_2_/Al_2_O_3_ molar ratio optimized for the requirements of specific applications. Although synthetic materials can be used for this purpose, it is not ideal, making the process more expensive.

Moreover, when using clay minerals such as kaolinite, it is necessary to convert them into metakaolinite through thermal treatment at temperatures between 500 and 800 °C. This treatment is favorable for increasing the reactivity of the mineral.^14^

Another fundamental component of AAMs is the alkaline activator, which is responsible for controlling the SiO_2_/Na_2_O ratio. This ratio also contributes to both the mechanical properties and the curing time. Commercial reagents are commonly used for this purpose, such as sodium hydroxide (NaOH), potassium hydroxide (KOH), calcium hydroxide [Ca­(OH)_2_], and sodium silicate (Na_2_SiO_3_). Although commercial activators confer favorable properties to the final products, their use increases the production costs. Alternatively, certain industrial waste materials—such as red mud, a byproduct of the Bayer process with high alkalinity, and carbide slag, a residue from the chlor-alkali industry—can serve as effective alkaline activators.^15,16^

Although the advantages of AAMs are significant, it is important to evaluate whether the raw material compositions, elements such as Na, Al, Si, and others, may be released into the environment when used in construction applications. Considering the benefits of AAMs and the essential components for their synthesis, the present study aims to produce an alkali-activated material from Amazon bauxite tailings—a source of aluminosilicate (kaolinite)—without converting it into metakaolinite, thereby reducing the energy required for synthesis and using only sodium hydroxide as the alkaline activator. Furthermore, this research evaluates the potential release of metals (Al, Cd, Na, Si, and Ti, among others) and metalloids (As) from the alkali-activated material into the environment.

## 2 Materials and Methods

### 2.1 Sampling

The sample employed in this study (ALB-1), representative of bauxite washing clay, was supplied by Mineração Paragominas S.A. (Pará, Brazil), in the municipality of Paragominas. To ensure its homogeneity, the material was collected directly from the pipeline transporting the residue to the disposal basins rather than from the basins themselves. Company personnel carried out sampling. Additionally, commercial-grade sodium hydroxide (NaOH) was employed as an alkaline activator.

### 2.2 Sample Characterization

#### 2.2.1 Mineralogical Composition

The sample was ground following the powder method to determine the mineralogical phases and subjected to X-ray diffraction. The analysis was carried out using a BRUKER diffractometer, model D2 PHASER (Bruker AXS GmbH, Karlsruhe, Germany), with a θ/θ goniometer, radius 141.1 nm, copper anode with a characteristic emission line of 1.54 Å/8.047 keV (Cu-Kα1), and a maximum power of 300 W (30 kV × 10 mA). The detector used was the Linear Lynxeye with a 5° 2θ aperture and 192 channels. All analyses were performed at the Laboratory of Mineralogy, Geochemistry, and Applications (LAMIGA-UFPA) of the Geosciences Institute at UFPA, Belém, Pará, Brazil.

#### 2.2.2 Chemical Composition

The chemical composition of sample ALB-1 was determined by X-ray fluorescence (XRF) spectrometry using fused pellets. The pellets were prepared at a ratio of 0.8 g of sample to 8 g of flux (lithium tetraborate), with the addition of approximately 5 mg (15%) of lithium bromide as a releasing agent. The analyses were carried out in a commercial laboratory.

Loss on ignition (LOI) was determined gravimetrically by calcining previously dried samples at 1000 °C.

#### 2.2.3 Fourier Transform Infrared (FTIR) Spectroscopy

This technique was used as a complementary analysis to the XRD results, aiding in the characterization of the raw samples. Approximately 0.0015 g of each sample was mixed with 2 g of potassium bromide (KBr, Merck, Darmstadt, Germany). The mixture was homogenized using an agate mortar and pestle. Pellets were then formed using 14 mm steel molds and pressed under an 8 Kbar pressure using a manual Specac 8 press. The analyses were performed using a Bruker Vertex 70 spectrometer (Bruker Optik GmbH, Ettlingen, Germany) in the spectral range of 400–4000 cm^–1^. This procedure was conducted at LAMIGA-UFPA of the Geosciences Institute at UFPA, Belém, Pará, Brazil.

#### 2.2.4 TG/DSC

Thermal analysis of the sample was performed using a NETZSCH STA 449 F5 Jupiter simultaneous thermal analyzer (NETZSCH-Gerätebau GmbH, Selb, Germany), equipped with a vertical cylindrical furnace, nitrogen flow of 50 mL/s, a heating rate of 10 °C/min, and a temperature range from 30 to 1100 °C. This procedure was carried out at LAMIGA-UFPA of the Geosciences Institute at UFPA, Belém, Pará, Brazil.

#### 2.2.5 Particle Size Determination

The particle size distribution of the ALB-1 sample was determined by laser diffraction granulometry using a Mastersizer 3000 optical system (Malvern Panalytical, Malvern, UK). For this analysis, the sample was dispersed in 100 mL of deionized water within the Hydro 2000S-A accessory (Malvern Panalytical). This volume was precisely adjusted to ensure optimal obscuration, a critical parameter for obtaining accurate particle size data. The experimental analyses were performed at the Instituto SENAI de Inovação em Tecnologias Minerais (ISI-TM), Belém, Pará, Brazil.

### 2.3 Geopolymer Synthesis, Hexagonal Paver Production, and Technological Tests

#### 2.3.1 Production of Hexagonal Pavers (25 cm × 25 cm)

Figure 1 illustrates the process of producing hexagonal pavers (ALK-1). A mixture containing 2.0 kg of ALB, 200 g of NaOH (to maintain a Na/Al molar ratio of 0.6), and 460 mL of water was prepared to obtain a solid-to-liquid ratio of 34.5%. For this process, a planetary mortar mixer (model 1.330.320, Solotest, Brazil) designed for cement and mortar preparation was used. The equipment comprises a 5 L stainless-steel bowl and a stainless-steel agitator (paddle) driven by a two-speed motor. In this study, the first (lower) speed was fixed during mixing. The molar ratio was established according to optimized synthesis procedures reported in a previous study.^17^ All components were transferred to a mortar and pestle mixer and homogenized for approximately 10 min. The resulting paste was then cast into hexagonal molds. Drying was performed in an electric oven at two temperatures: 40 °C for 1 week and 50 °C for 3 weeks.

**Figure 1:**
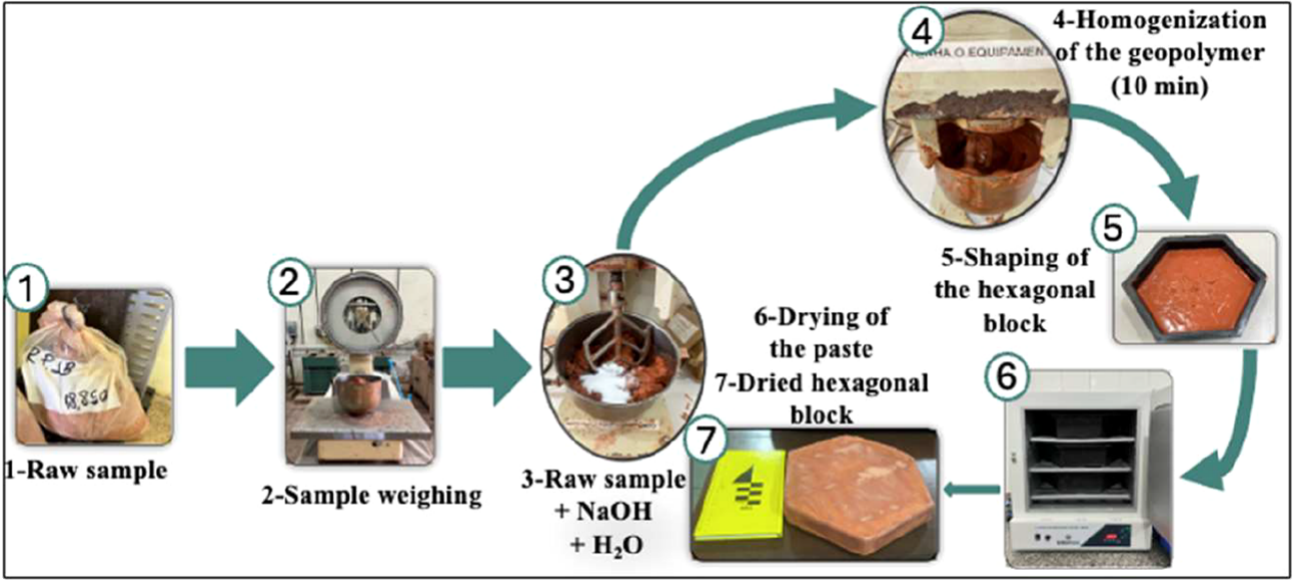
Schematic representation of the preparation process for the hexagonal block.

### 2.4 Geopolymer Testing

All technological tests and leaching assays were performed in triplicate using specimens with dimensions of 20 × 10 × 10 mm prepared from the hexagonal block (Figure 2). The selection of this nonstandard geometry represents a necessary adaptation for the characterization of a novel geopolymer product at a laboratory scale. It is increasingly recognized that the mechanical properties of geopolymers are subject to significant size and shape effects and that testing protocols designed for conventional materials like cement or ceramics are often inadequate for these advanced binders.^18^

**Figure 2:**
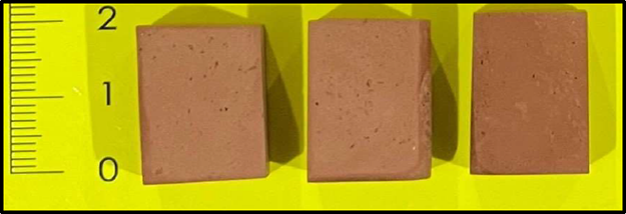
Test specimens used in technological evaluations.

#### 2.4.1 Water Absorption and Apparent Porosity

These parameters were evaluated following the ASTM C20-00 standard.^19^

#### 2.4.2 Compressive Strength

Compressive strength tests were conducted using a universal testing machine, model 300/15 kN, Servo-plus Evolution from MSTEST (MSTest, São Paulo, Brazil), with a loading rate of 1 MPa/s. The test started at 1 N and ended at 30% of the maximum load. The test was performed at the Concrete Laboratory of the School of Architecture at UFPA, Technology Institute at UFPA, Belém, Pará, Brazil.

#### 2.4.3 Leaching Test and Analysis of Leachates

The leaching test was performed using a method adapted from the Brazilian Association of Technical Standards-ABNT 10005 (NBR 10005; Sampling of liquid effluents—Procedure. ABNT: Rio de Janeiro, Brazil, 2004).^20^ The solution used was 0.1 M HNO_3_. The test involved immersing triplicate specimens in the acid solution for 7 (experiments 1, 2, and 3), 14 (experiments 4, 5, and 6), 21 (experiments 7, 8, and 9), and 28 days (experiments 10, 11, and 12). At the end of each cycle, the solution was collected and replaced with a fresh one to proceed to the next cycle. The leachates were analyzed by using inductively coupled plasma optical emission spectrometry (ICP-OES). Based on the sample composition and the NaOH reagent, the analytes selected were Al, As, Ca, Cd, Cr, Fe, Mg, Mn, Na, Ni, Pb, Si, and Ti. The analytical curve was prepared in an acidic medium (2% v/v HNO_3_), with concentrations of 0, 2, 4, 6, 8, and 10 mg/L. The method of analyte addition and recovery was used to validate the measurements.

#### 2.4.4 Scanning Electron Microscopy (SEM)

Images were obtained from the ALK-1 sample using a HITACHI TM-3000 scanning electron microscope, coupled with an energy-dispersive spectrometer (EDS), SWIFT ED-3000, from Oxford Instruments (Oxford, UK). The analyses were performed at LAMIGA-UFPA of the Geosciences Institute at UFPA, Belém, Pará, Brazil.

## 3 Results

### 3.1 Raw Sample

#### Mineralogy by XRD, Chemical Composition, FTIR Spectroscopy, Thermal Analysis (TG/DSC), and Particle Size Distribution

Figure 3 shows the X-ray diffractogram of sample ALB-1. It can be observed that the sample is composed of characteristic peaks of minerals, such as kaolinite (46%), gibbsite (34%), Al-goethite (10%), hematite (7%), and anatase (2%). This mineralogical composition is well-established for bauxite washing clay samples. In the present study, which focuses on the synthesis and application of geopolymer, one of the most relevant aspects regarding the sample’s mineralogy is the predominance of kaolinite, the main aluminosilicate mineral used for geopolymer production.^21–23^

**Figure 3:**
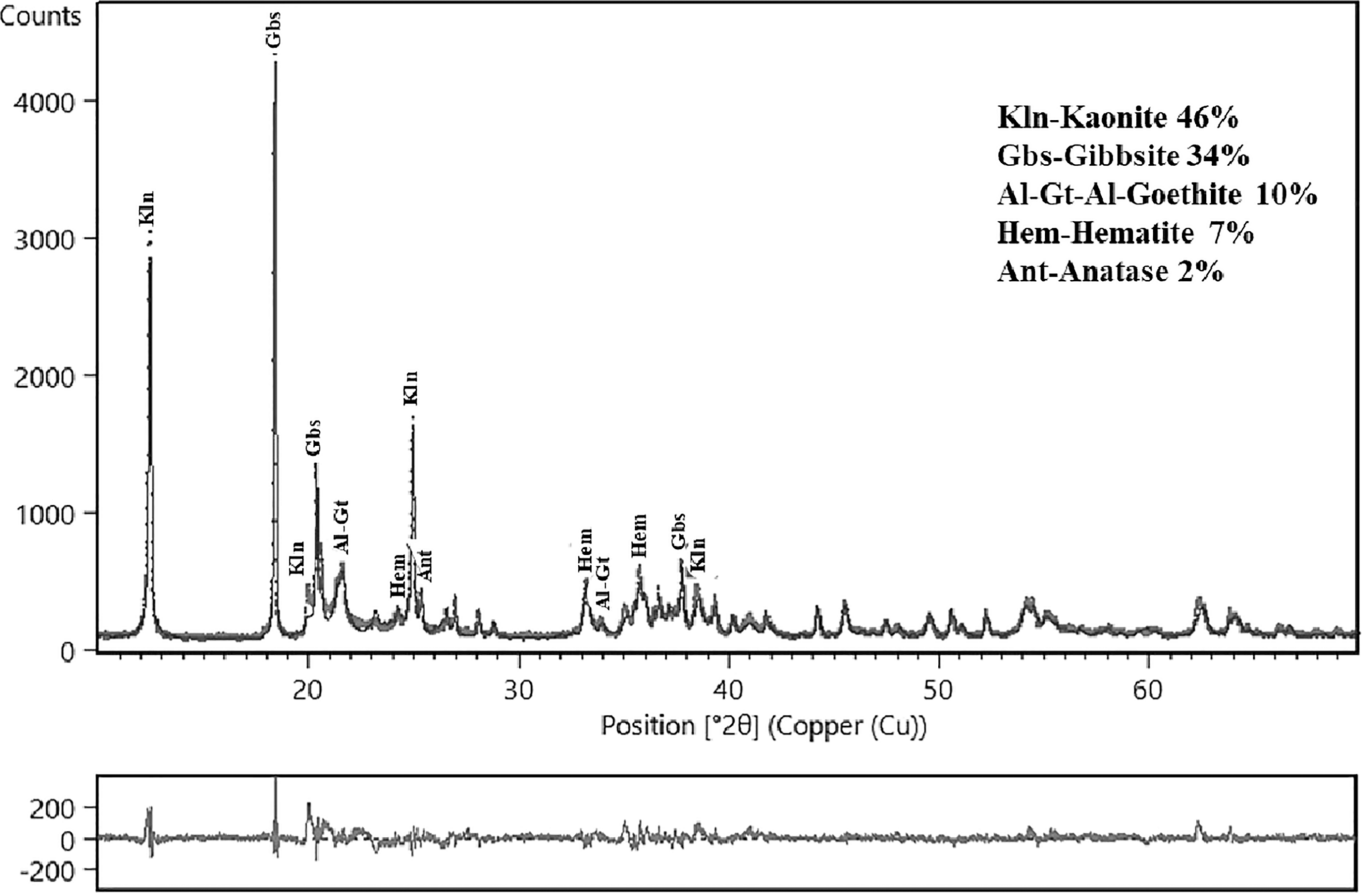
X-ray diffraction pattern of sample ALB-1 refined using the Rietveld method. GOF: 1.61, *R*_wp_: 11.87.

Table 1 shows the chemical composition of the ALB-1 sample. The major constituents are Al_2_O_3_, SiO_2_, and Fe_2_O_3_, which together represent more than 80% of the total oxide composition. This content is associated with the mineralogy of the sample. Al_2_O_3_ is present in kaolinite, gibbsite, and Al-goethite; SiO_2_ is essentially from kaolinite, while Fe_2_O_3_ is derived from hematite and goethite. The remaining percentage relates to the LOI (18.73%) and a minor quantity of other compounds.

**Table 1:** Chemical Composition of Sample ALB-1 by X-ray Fluorescence (XRF)

compound	content (% wt)
Na_2_O	0.03
MgO	0.12
Al_2_O_3_	41.76
SiO_2_	25.74
P_2_O_5_	0.05
SO_3_	
K_2_O	0.01
CaO	
TiO_2_	2.07
Fe_2_O_3_	12.59
LOI	18.73
Sum	101.25

The FTIR spectrum is shown in Figure 4. The absorption bands at 3437, 3525, 3620, and 3695 cm^–1^ are associated with the O–H bond present in kaolinite, gibbsite, and water.^21^ The band at 1635 cm^–1^ corresponds to the vibrational mode of the water molecules. The absorption near 1100 cm^–1^ is characteristic of the Si–O stretching bond in kaolinite. The band at 1010 cm^–1^ is also attributed to kaolinite and corresponds to the symmetric stretching of the Si–O–Si bond. The bands at 468 and 750 cm^–1^ are related to symmetric stretching of the Al–O bond. The bands at 912 and 796 cm^–1^ are assigned to the angular deformation of the Al–OH bond and translational vibration of the −OH group in gibbsite, respectively. The absorption at 538 cm^–1^ is related to the angular deformation of the Fe–O bond in hematite.

**Figure 4:**
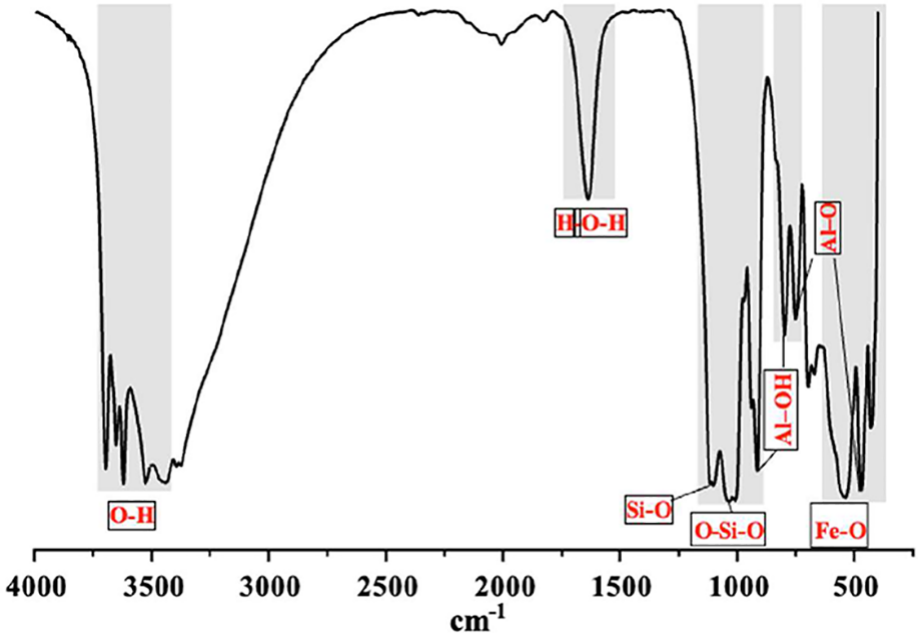
FTIR spectra of sample ALB–1.

Figure 5 presents the TG/DSC curves of the ALB-1 sample. The curves show characteristic events of the minerals identified by XRD. Three endothermic events and one exothermic event were observed. The first endothermic peak at 283 °C corresponds to the release of structural water from the gibbsite. The second peak, at 353 °C, confirms the presence of goethite, characterized by the release of constitutional water. The third endothermic event at 503 °C is associated with the dehydroxylation of kaolinite, leading to the formation of metakaolinite.^24,25^ The only exothermic event, observed at 980 °C, corresponds to the crystallization of mullite.^24^

**Figure 5:**
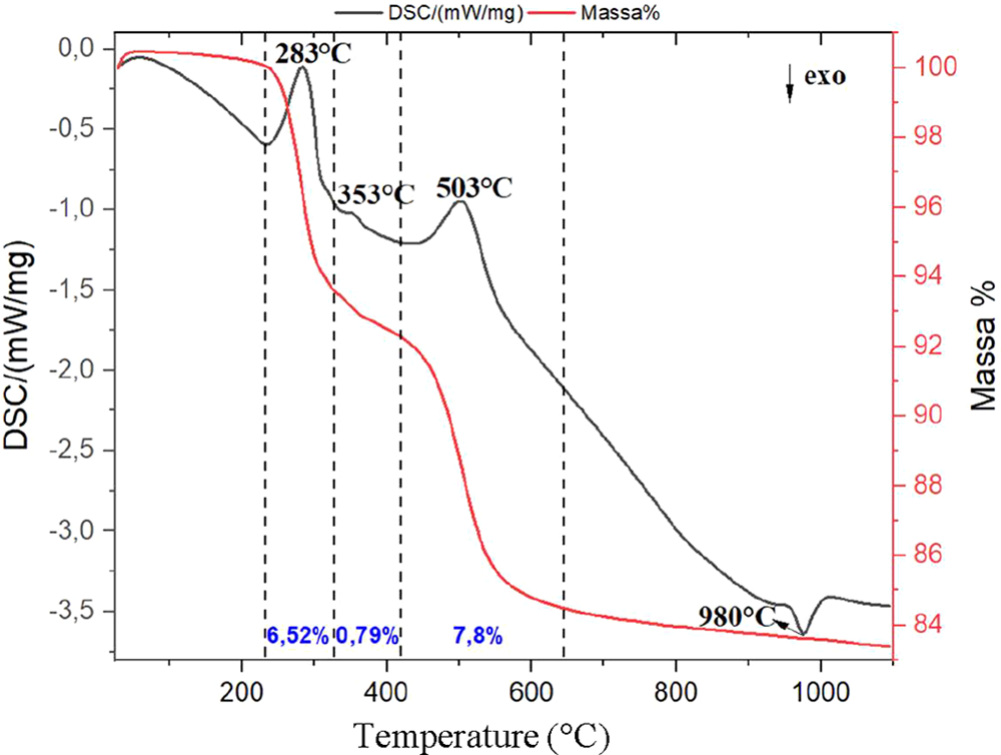
TG/DSC curves of sample ALB-1.

As shown in Figure 6, fine particles were predominant in the ALB-1 sample. The particle size distribution revealed a D10 of 3.22 μm, a D50 of 11.7 μm, and a D90 of 49.7 μm, indicating that 10, 50, and 90% of the particles were below these sizes. The predominance of fine particles, evidenced by the relatively low D50 value, is particularly favorable for geopolymerization, as particle fineness plays a crucial role in geopolymer binders’ reactivity and microstructural development.^26^ The enhanced reactivity associated with finer particle fractions can be attributed to the increased specific surface area available for dissolution in the alkaline activating solution, which accelerates the release of reactive aluminosilicate species and promotes the formation of a denser N–A–S–H gel network.^26,27^ This particle size distribution is expected to contribute positively to both the mechanical performance and the uniformity of the geopolymer microstructure.

**Figure 6:**
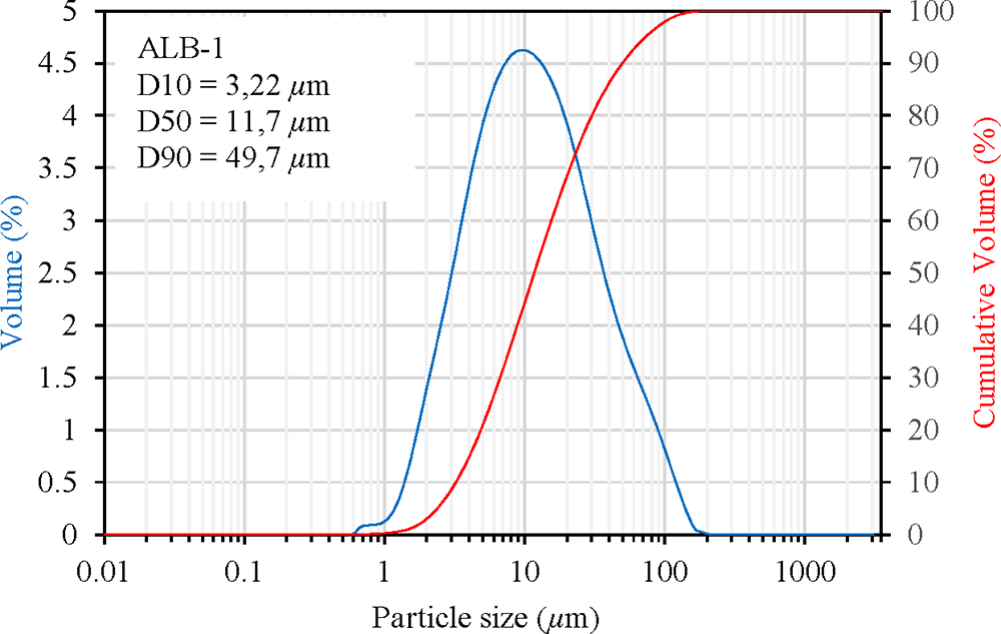
Particle size analysis of the ALB-1 sample.

### 3.2 Alkali-Activated Specimen (ALK-1)

#### 3.2.1 Crystalline and Amorphous Phases after XRD

The mineralogical composition of the block (ALK-1) is shown in Figure 7. The results indicate the persistence of all minerals present in the raw sample: kaolinite, gibbsite, hematite, goethite, and anatase. However, their relative contents significantly decreased compared to those of the original sample. Kaolinite, for example, which initially constituted approximately 46% of the sample, was reduced to 4.3%. This reduction confirms that most of the kaolinite reacted with the NaOH solution, resulting in the formation of zeolite ZK-14 (1.5%), sodalite (13.4%), cancrinite (7%), and amorphous phases (25.9%).^21,24^ This composition suggests that the resulting alkali-activated material can be classified as a hybrid product composed of both crystalline phases (ZK-14 and cancrinite) and amorphous phases. The presence of crystalline phases contributes to improved refractory properties.^28^ Additionally, the presence of sodium carbonate suggests that part of the product reacted with atmospheric CO_2_. This phenomenon can be mitigated by curing the material in a controlled humidity environment.

**Figure 7:**
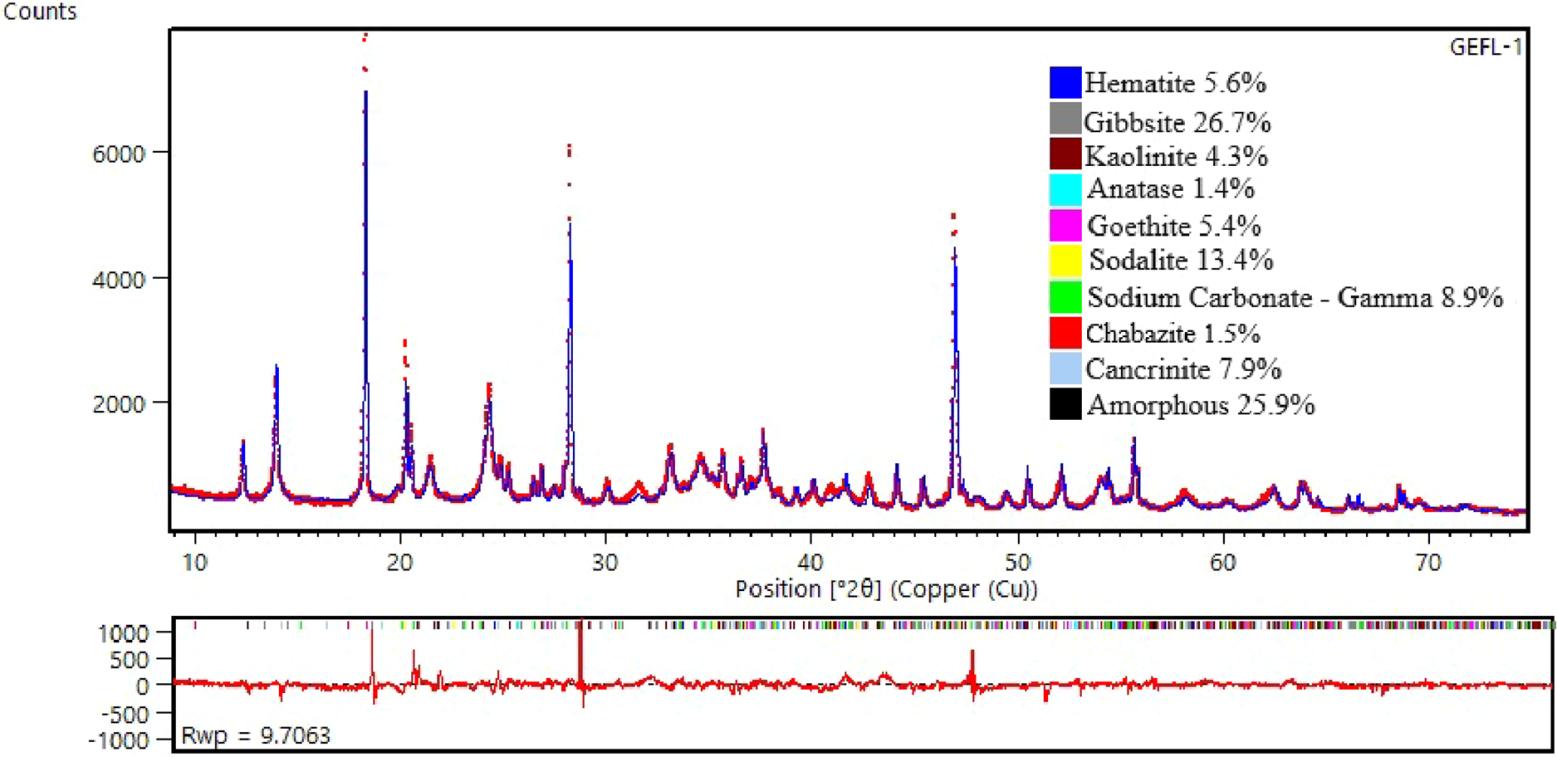
X-ray diffraction pattern of sample ALK-1 refined using the Rietveld method: GOF: 2.17, *R*_wp_: 9.70.

#### 3.2.2 Microscopical Texture Features by SEM

The microstructure of the geopolymer, as observed by SEM, is shown in Figure 8. The presence of an amorphous gel phase is evident, indicating successful geopolymer formation. Some larger particles suggest an incomplete reaction (Figure 8a). In Figure 8b, rod-like structures are observed, likely corresponding to cancrinite crystals,^29,30^ as well as polycrystalline porous agglomerates that may be related to sodalite.^28,31^ The formation of needle-like structures may result from the later stages of the transitional gel phase, possibly indicating the progressive crystallization or organization of the gel components.^6^ The crack density in this higher magnification image appears moderate, with microcracks potentially serving as stress concentration points that could affect the material’s fracture behavior.^32,33^ The interconnected gel network demonstrates good coverage around the crystalline phases, which is essential for load transfer and overall mechanical integrity.^34,35^

**Figure 8:**
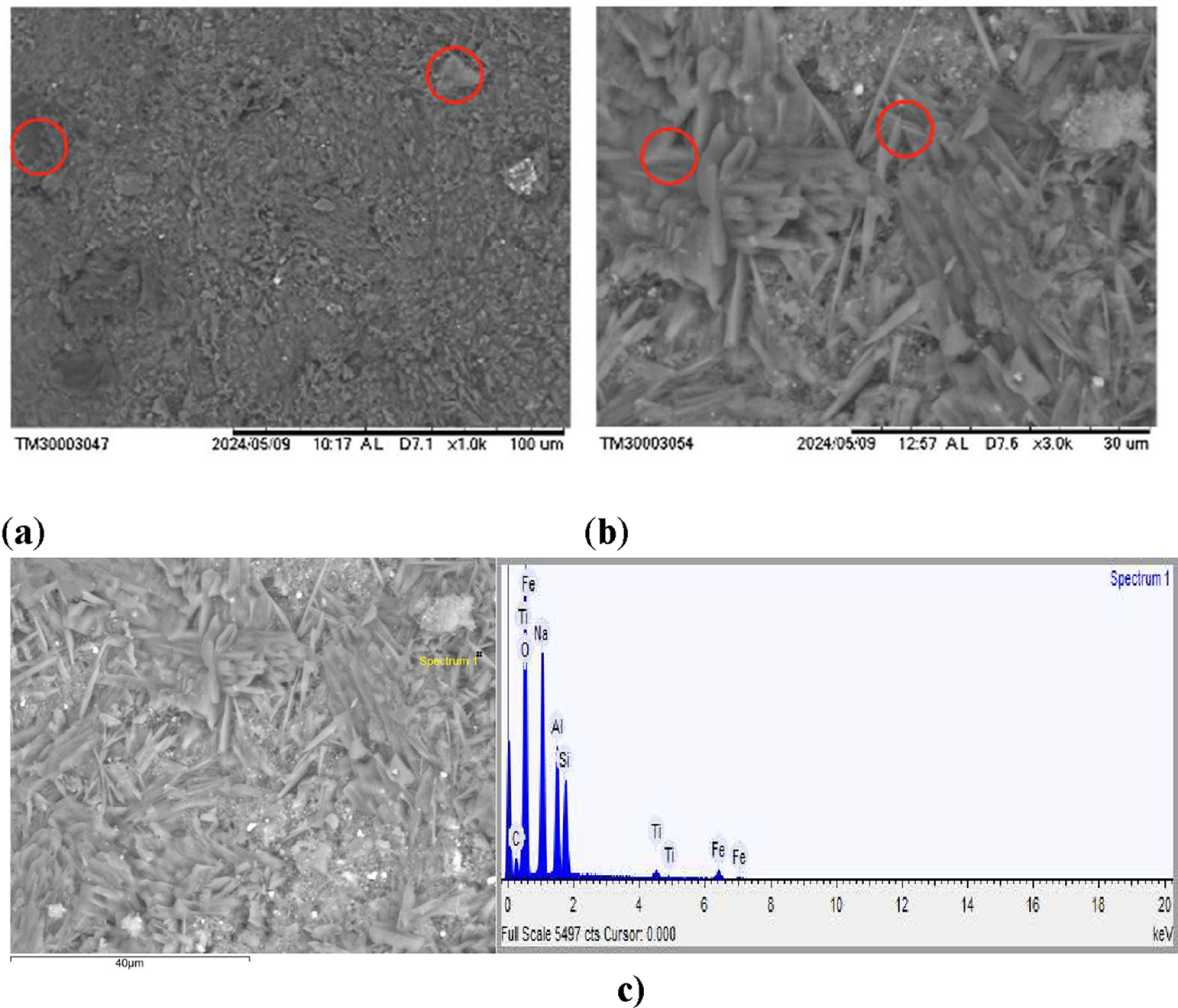
Scanning electron micrographs of alkali-activated material (ALK-1), showing (a) larger grains (∼100 μm), (b) finer grains (∼30 μm), and (c) EDS of needle-like structures.

The microstructure of the geopolymer, as observed by SEM, is shown in Figure 8. The presence of an amorphous gel phase is evident, indicating a successful geopolymer formation. Some larger particles suggest an incomplete reaction (Figure 8a). The microstructure exhibits a relatively porous nature with visible microcracks (marked by red circles), which are commonly associated with shrinkage during curing or incomplete gel densification.^36,37^ However, the gel coverage appears to be reasonably uniform across most regions, suggesting adequate geopolymerization despite the presence of unreacted particles.

The EDS results from the needle-like (Figure 8c) structures indicate that the material is mainly composed of Al, Si, and Na, along with minor amounts of Ti and Fe. These elements are likely associated with the presence of the sodalite mineral.

#### 3.2.3 Technological Properties

Figure 9 presents the results for the apparent density (AD), apparent porosity (AP), water absorption (WA), and compressive strength (CS). The geopolymer exhibited an apparent density of 1.60 g/cm^3^ ± 0.04, indicating good compaction with the defined NaOH and water ratios. Apparent porosity was 29.78% ± 1.06. Water absorption was 18.61% ± 0.25, which is favorable when compared to the standard limits for tiles and bricks (20–22%). The compressive strength reached 25.83 MPa ± 1.33, which is considered high, especially considering that the ALB-1 sample was not thermally treated to convert kaolinite into metakaolinite, and no external source of SiO_2_ was added during synthesis.

**Figure 9:**
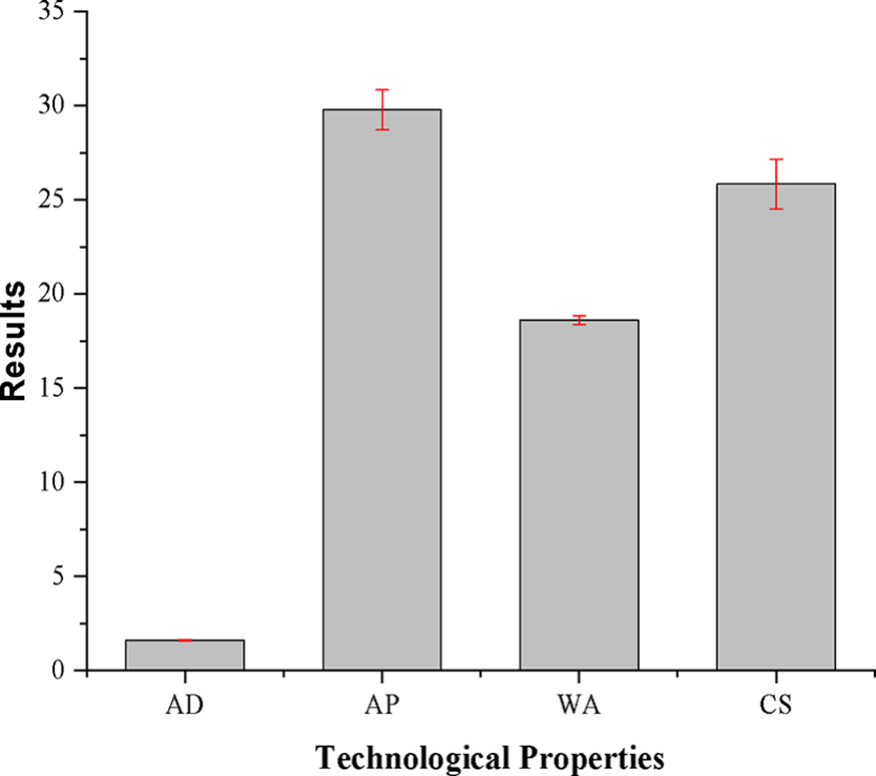
Physical tests performed on the geopolymer.

#### 3.2.4 Leaching Test

The concentrations of leached elements after 7, 14, 21, and 28 days are shown in Table 2. The concentrations of As, Ca, Cr, Fe, Mg, Mn, Ni, and Pb were below the detection limit of the equipment, indicating that if released, these elements are present in concentrations lower than 0.001 mg/L (e.g., the detection limit for Pb). In contrast, Al, Cd, Na, Si, and Ti were detected in all leaching cycles. Cadmium concentrations remained constant throughout the cycles, ranging from 0.02 to 0.03 mg/L. Titanium was detected at only 7 days (5.33 mg/L). Silicon was released along all cycles with concentrations of 3.93 mg/L (7 days), 66.52 mg/L (14 days), 29.20 ± 0.38 mg/L (21 days), and 46.76 ± 0.08 mg/L (28 days). Aluminum concentrations ranged from 26.27 ± 16.24 mg/L (21 days) to 36.34 ± 10.16 mg/L (28 days), with the highest release occurring at the longest testing period. Sodium showed the highest concentrations, ranging from 1375.31 ± 9.74 mg/L (7 days) to 513.77 ± 11.30 mg/L (28 days), with the highest leaching at the shortest time.

**Table 2:** Element Concentrations in Leachate Extracts (mg/L)

sample	Al 396.1	Cd 228.8	Na 589.5	Si 212.4	Ti 334.9
1	36.34 ± 10.16	0.03 ± 0.02	513.77 ± 11.30	54.22 ± 0.91	LOD
2	181.76 ± 4.82	0.03 ± 0.04	548.98 ± 2.52	46.76 ± 0.08	LOD
3	LOD	0.01 ± 0.01	665.44 ± 3.16	54.40 ± 2.96	LOD
4	73.46 ± 11.00	LOD	648.70 ± 20.69	29.20 ± 0.38	LOD
5	26.27 ± 16.24	0.04 ± 0.01	887.14 ± 29.31	35.37 ± 1.53	LOD
6	61.09 ± 21.55	0.02 ± 0.03	692.37 ± 23.98	26.06 ± 1.96	LOD
7	118.64 ± 9.95	0.02 ± 0.01	1045.72 ± 62.61	69.83 ± 4.66	LOD
8	36.34 ± 10.16	0.02 ± 0.02	741.65 ± 7.68	63.21 ± 3.62	LOD
9	33.80 ± 15.149	0.01 ± 0.01	836.14 ± 7.80	64.81 ± 2.46	LOD
10	LOD	0.08 ± 0.04	1375.31 ± 9.74	3.94 ± 2.26	LOD
11	LOD	0.03 ± 0.02	3517.44 ± 307.24	30.82 ± 4.60	6.10 ± 0.79
12	LOD	0.03 ± 0.02	1748.55 ± 41.44	LOD	4.60 ± 0.52

## 4 Discussion

### 4.1 Physical Properties

The synthesis parameters adopted in this study—including water content, NaOH concentration, and curing time and temperature—proved to be effective for producing alkali-activated pastes with desirable technological properties. Specifically, the materials demonstrated good compaction, adequate density, and low values of water absorption (WA) and apparent porosity (DA), indicating a well-structured matrix.

The compressive strength results align with findings from other studies^9,38^ that utilized NaOH and alternative alkaline activators. Some studies have reported compressive strengths of up to 12.2 MPa after 90 days of curing with NaOH as the activator,^5^ while others have reached values as high as 32.97 MPa.^39^ The compressive strength obtained in this study is considered adequate, suggesting that the product could be used as an additive for cement or even as a partial substitute for concrete.^40,41^ When compared to previous studies utilizing similar bauxite tailings,^21^ the compressive strength result obtained in this work is notably positive. The high strength reported by these authors, which ranged from 3.2 to 69.7 MPa, was generally contingent upon the incorporation of supplementary cementitious materials, such as ground granulated blast furnace slag (GGBS). Furthermore, the alkaline activation in those studies frequently involved the use of sodium silicate (Na_2_SiO_3_), a component that significantly impacts the final cost of the geopolymer product. The superior performance achieved in this study, potentially with a more simplified or lower-cost formulation, demonstrates a promising advancement in optimizing the production of geopolymers derived from bauxite tailings. For instance, a study on geopolymers derived from 100% sewage sludge ash (SSA) reported a maximum compressive strength of 21.3 MPa under optimized curing conditions.^42^ Similarly, research on solid backfilling materials based on carbonated coal-based waste (CCBW) showed a maximum uniaxial compressive strength decrease of only 1.62% after 28 days of immersion in mine water, indicating a high initial strength, though the exact value was not explicitly stated in the abstract.^43^ The fact that our bauxite tailing-based geopolymer, activated solely with NaOH, surpasses the strength of the SSA-based geopolymer and aligns with the performance of more complex CCBW-based materials underscores the high reactivity and suitability of the bauxite tailings precursor. This performance is particularly relevant for applications where moderate to high strength is required, such as nonstructural concrete elements or stabilization of mine waste, without the need for costly additives like metakaolin or sodium silicate, which were often necessary to achieve viable strength in other waste-based systems.^42^

For our study, this performance can be attributed, in part, to the presence of zeolitic phases in the precursor, which are known to enhance durability and weathering resistance.^44^ This crystalline phase, which forms concurrently with the amorphous geopolymeric gel, acts as a microaggregate that contributes significantly to the densification and mechanical integrity of the final product. As supported by microstructural evidence (SEM images showing a dense interlocked structure), the zeolitic crystals precipitate within the pore network, effectively reducing the overall porosity and increasing the stiffness of the matrix. Within the context of using a bauxite residue as the aluminosilicate source, the data suggest that costly additives, such as commercial silica or sodium silicate, may be unnecessary for achieving satisfactory mechanical performance.

Moreover, the physical performance observed is particularly noteworthy when compared to the literature that reports high-strength materials typically based on metakaolin synthesized under elevated temperatures. In contrast, the approach adopted here eliminated the need for thermal activation and still resulted in a structurally competent product,^14,45–47^ highlighting its potential for more sustainable and cost-effective applications.

### 4.2 Leaching Test

Elevated release levels of certain elements may suggest a moderate dissolution of the geopolymeric gel—mainly composed of Al, Si, and Na—under mildly acidic conditions. This elemental release may also be influenced by the concentration of the alkali activator, which increases the pH of the medium and contributes to the leaching of these elements, as previously reported by.^48^ Similar to other studies, this product does not release toxic metals, such as lead, chromium, and arsenic.^41^ Based on the percentage values, none of the released elements exceeded 1% of leaching (Table 1), which demonstrates the chemical stability of the geopolymer matrix relative to the composition of the initial material. The observed leaching behavior, particularly the high initial release of Na, is a common characteristic in geopolymer systems activated with high concentrations of NaOH.^43^ This trend is primarily attributed to the dissolution of an unreacted alkaline activator and the subsequent stabilization of the geopolymeric matrix over time, which physically entraps the mobile ions. Comparative studies on geopolymer-based solid backfilling from CCBW also report that the leaching concentration of Na is the highest, followed by Si and Al.^43^ In that study, the carbonation process was found to reduce the leaching of Na, Al, and Si by 15.76, 32.86, and 3.21%, respectively, by forming a denser pore structure. Furthermore, the leaching of Si and Al, which are the backbone elements of the geopolymer structure, is a critical indicator of the material’s long-term stability. The Si and Al concentrations observed in our study are comparable to the general trends reported in other waste-based geopolymers, where the dissolution of these elements is facilitated by the alkaline environment.^42,43^ The relatively high leaching of these elements, particularly Si, suggests that while the material is structurally sound, its application in highly sensitive aquatic environments may require further optimization, such as the incorporation of Supporting Information or a post-treatment process like carbonation, to meet the strictest international regulatory limits for inert waste.^42^

Although the concentrations of Al, Na, and Si exceeded the regulatory limits established by CONAMA Resolutions 454/12 and 420/09, parameters such as temperature, simulated rainfall, and granulometry must be evaluated, as these elements may be released into the environment depending on local application conditions. For international standards such as the USEPA TCLP (Method 1311) and EN 12457, the maximum permissible limits are well-defined for heavy metals. The TCLP establishes regulatory levels of 5.0 mg/L for arsenic, chromium, and lead, while EN 12457 sets limits of 0.5 mg/kg for inert waste landfills. However, these standards do not consider aluminum, silicon, and sodium as priority pollutants (Table 3). In this case, other aspects must be carefully evaluated, including the specific application context and site-specific environmental conditions.^49^ Furthermore, the potential impact on human health should be carefully assessed.^41,50,51^

**Table 3:** Al, Na, and Si Concentrations in Leachate Extracts % (w/w)

element	concentration (mg/L)	% (w/w)
Si	3.93 (7d)/66.52 (14d)/29.20 ± 0.38 (21d)/46.76 ± 0.08 (28d)	0.000393%/0.006652%/0.002920 ± 0.000038%/0.004676 ± 0.000008%
Al	26.27 ± 16.24 (21d)/36.34 ± 10.16 (28d)	0.002627 ± 0.001624%/0.003634 ± 0.001016%
Na	1375.31 ± 9.74 (7d)/513.77 ± 11.30 (28d)	0.137531 ± 0.000974%/0.051377 ± 0.001130%

## 5 Conclusions

The bauxite washing residue sample is predominantly composed of aluminum- and silica-rich minerals (kaolinite and gibbsite), which are favorable for geopolymer synthesis.

The direct geopolymerization process, carried out without thermal pretreatment or external silica addition, demonstrated promising technological potential, as evidenced by the low water absorption and substantial mechanical strength. These characteristics suggest that the resulting product could serve as an excellent and ecofriendly alternative for applications in the construction sector.

Regarding the release of metals and metalloids, the most prominent elements detected were Al, Si, and Na—major constituents of the geopolymeric matrix. The leaching behavior of these elements requires further investigation to ensure environmental safety. Additionally, a broader range of parameters must be assessed to fully understand the conditions that may trigger leaching of all analyzed elements.

Based on the results presented, the material studied shows promising potential for use in paving block production pending further investigation into environmental implications.

## Data Availability

The original contributions presented in this study are included in the article. Further inquiries can be directed to the corresponding author.
